# Combining behavioural activation with physical activity promotion for adults with depression: findings of a parallel-group pilot randomised controlled trial (BAcPAc)

**DOI:** 10.1186/s13063-015-0881-0

**Published:** 2015-08-20

**Authors:** Claire Pentecost, Paul Farrand, Colin J. Greaves, Rod S. Taylor, Fiona C. Warren, Melvyn Hillsdon, Colin Green, Jo R. Welsman, Kat Rayson, Philip H. Evans, Adrian H. Taylor

**Affiliations:** Complex Interventions Research Group, University of Exeter Medical School, South Cloisters 1.32, Heavitree Road, Exeter, EX1 2LU UK; Clinical Education Development and Research, Psychology, Washington Singer Laboratories, University of Exeter, Perry Road, Exeter, EX4 4QG UK; University of Exeter Medical School, St Luke’s Campus, Heavitree Road, Exeter, EX1 2LU UK; Sport & Health Sciences, University of Exeter, St Luke’s Campus, Heavitree Road, Exeter, EX1 2LU UK; Lived Experience Group, Mood Disorders Centre, Psychology, Washington Singer Laboratories, University of Exeter, Perry Road, Exeter, EX4 4QG UK; Plymouth University Peninsula Schools of Medicine & Dentistry, N6 ITTC Building, Plymouth Science Park, Derriford, Plymouth, PL6 8BU UK

**Keywords:** Behavioural activation, IAPT, Low-intensity Cognitive Behavioral Therapy, Mixed methods, Physical activity, Pilot RCT, Qualitative, Self-help, Trial

## Abstract

**Background:**

Depression is associated with physical inactivity, which may mediate the relationship between depression and a range of chronic physical health conditions. However, few interventions have combined a psychological intervention for depression with behaviour change techniques, such as behavioural activation (BA), to promote increased physical activity.

**Methods:**

To determine procedural and clinical uncertainties to inform a definitive randomised controlled trial (RCT), a pilot parallel-group RCT was undertaken within two Improving Access to Psychological Therapies (IAPT) services in South West England. We aimed to recruit 80 adults with depression and randomise them to a supported, written self-help programme based on either BA or BA plus physical activity promotion (BAcPAc). Data were collected at baseline and 4 months post-randomisation to evaluate trial retention, intervention uptake and variance in outcomes to inform a sample size calculation. Qualitative data were collected from participants and psychological wellbeing practitioners (PWPs) to assess the acceptability and feasibility of the trial methods and the intervention. Routine data were collected to evaluate resource use and cost.

**Results:**

Sixty people with depression were recruited, and a 73 % follow-up rate was achieved. Accelerometer physical activity data were collected for 64 % of those followed. Twenty participants (33 %) attended at least one treatment appointment. Interview data were analysed for 15 participants and 9 study PWPs. The study highlighted the challenges of conducting an RCT within existing IAPT services with high staff turnover and absences, participant scheduling issues, PWP and participant preferences for cognitive focussed treatment, and deviations from BA delivery protocols. The BAcPAc intervention was generally acceptable to patients and PWPs.

**Conclusions:**

Although recruitment procedures and data collection were challenging, participants generally engaged with the BAcPAc self-help booklets and reported willingness to increase their physical activity. A number of feasibility issues were identified, in particular the under-use of BA as a treatment for depression, the difficulty that PWPs had in adapting their existing procedures for study purposes and the instability of the IAPT PWP workforce. These problems would need to be better understood and resolved before proceeding to a full-scale RCT.

**Trial registration:**

ISRCTN74390532. Registered on 26 March 2013.

**Electronic supplementary material:**

The online version of this article (doi:10.1186/s13063-015-0881-0) contains supplementary material, which is available to authorized users.

## Background

The experience of reduced activities of daily living, including physical activity (PA), is a common feature of depression [1]. People with depression are also at greater risk of comorbid conditions such as diabetes [2], stroke [3] and obesity [4]. PA can enhance both physical [5] and mental health [6, 7] for people with depression, and it has been shown to reduce the risk of depressive relapse [8] after recovery. Despite this evidence, in a recent systematic review of the effectiveness of psychological therapies for depression, none of the 204 trials evaluated measured PA [9], suggesting a lack of interest in targeting physical and mental health outcomes together and an implicit or explicit denial that changes in PA can mediate further improvements in mood. Although there is increasing interest in integrated care, little evidence exists regarding the best way to promote PA within routine psychological therapies.

In England, the Improving Access to Psychological Therapies (IAPT) programme [10] seeks to implement National Institute of Health and Care Excellence guidelines recommending a number of evidence-based psychological therapies for depression and anxiety. Patients self-refer, or are referred by, their general practitioner (GP) to IAPT services. Behavioural activation (BA) [11] is one such low-intensity psychological therapy for people with depression. Adopting a systematic and graded approach to increasing daily activities, BA targets the behavioural inertia and avoidance that often accompany depression. This helps to overcome sources of negative reinforcement that maintain avoidance whilst exposing the patient to sources of positive reinforcement within the environment [11]. Although its aim is to increase daily activities, BA does not conventionally employ techniques associated with PA promotion [12]. However, a focus on improving PA within BA could potentially be achieved with only a minor shift in emphasis. In particular, this can be accomplished through selective reinforcement of activities that require greater energy expenditure, alongside the use of other behaviour change techniques specifically targeted at promoting PA [12].

BA delivered within IAPT services is client-centred in a variety of ways. Self-determination theory (SDT) [13] highlights the importance of the human needs of feeling competent, in control and connected with others, and it fits well within the delivery of BA in IAPT services. SDT has also been the basis for PA interventions [14]. Developing behavioural self-regulatory skills to become more physically active [13, 6] could also help to overcome limitations encountered when trying to encourage people with symptoms of low confidence and low energy associated with depression.

This pilot parallel-group randomised controlled trial (RCT) was carried out consistent with the Medical Research Council Framework for the Development and Evaluation of Complex Interventions [15]. The overarching aim was to address methodological, procedural and clinical uncertainties to inform the conduct of a definitive RCT to assess the clinical effectiveness and cost-effectiveness of BA (a written self-help programme based on BA supported by a psychological wellbeing practitioner [PWP]) compared with the same BA written self-help programme informed by SDT combined with PA promotion supported by a PWP (BAcPAc). In this pilot trial, we sought to (1) assess the feasibility of recruiting participants from two IAPT services, (2) assess the feasibility of data collection procedures, (3) estimate variance in outcomes to inform future sample size, (4) estimate resource use and related costs associated with intervention delivery and (5) assess the feasibility and acceptability of the intervention.

## Methods

### Design

This pilot trial used a randomised trial design and mixed methods [16] described previously [17]. We sought to address the objectives by (1) reporting numbers of participants invited, screened, randomised and uptake to randomised allocation and those completing follow-up, exploring experiences of PWPs inviting participants and exploring understanding and acceptability of study recruitment procedures with participants; (2) reporting completed data collection at baseline and follow-up; (3) reporting descriptive statistics related to the planned outcomes of a future study; (4) assessing variability in the number, length and frequency of support sessions provided by PWPs; (5) exploring views and experiences of delivering the intervention with PWPs and participants who received at least one treatment session, and collecting recorded sessions between PWPs and participants to measure fidelity to the intervention protocol.

### Participants and recruitment

Participants were recruited from two IAPT services in South West England between 8 April 2013 and 28 January 2014. Informed consent was obtained for all participants. One IAPT service served a city location (site 1) and the other served several towns and villages (site 2). On the basis of service data collected 6 months before recruitment, it was predicted that approximately 240 people per month (70 % of all referrals) would enter each service with depression. It was anticipated that 13 % (*n* = 31) would respond, of whom 60 % (*n* = 19) per month would have a clinical diagnosis of depression and be entered into the study. At site 1, recruitment would be lower, with 4 of 19 service PWPs involved with inviting potential participants. Our target was to recruit 80 participants in a 10-month period for this pilot study.

Eligible participants were aged 18 years and older, had a current primary diagnosis of depression confirmed using the structured diagnostic Clinical Interview Schedule–Revised (CIS-R) [18] and scored between 10 and 23 on the Patient Health Questionnaire (PHQ-9) [19], reflecting moderate to moderate-severe depression. Eligible participants were also required to confirm that they could walk continuously and unaided for 5 minutes. Individuals were excluded if they (1) were currently receiving formal psychotherapy, (2) had been diagnosed with a severe and enduring mental health problem, (3) were already doing at least 30 minutes of moderate intensity PA on 5 or more days per week, (4) scored 2/3 on PHQ-9 [19] suicide risk question 9, (5) had a change in antidepressant medication in the month before recruitment, (6) had current substance or alcohol addiction, (7) were unable to use written self-help materials in English or (8) were currently involved in another research study.

The recruitment pathways at site 1 (Additional file 1) and site 2 (Additional file 2) were agreed following several meetings with site managers and study PWPs. Four PWPs were recruited to support the study intervention in each site, two of whom were randomly allocated to each study arm. In site 1, all PWPs were trained to hand out a study invitation pack (invitation letter, information sheet, consent form with a reply slip and stamped, addressed envelope) to all potential participants scoring between 10 and 23 on the PHQ-9, suggesting potential depression, and suitable for low-intensity psychological therapy based on BA treatment in accordance with normal service criteria. Alongside standard service information, all potential participants who were referred or self-referred into the service at site 2 were sent a study invitation pack. After receiving full written consent, the researcher assessed potential eligibility. The eligibility assessment was then completed, including use of the CIS-R [18] to confirm depression. If depression was confirmed, participants completed the baseline assessment and were then randomised using a web-based minimisation programme provided by the Peninsula Clinical Trials Unit [20] to ensure concealment of allocation to the study arm.

Additional efforts were undertaken to ensure research staff were kept blinded to participant allocation at all times during the study. Throughout the trial, participants were reminded not to disclose the PWP to whom they had been randomised, and PWPs were reminded not to disclose the treatment arm to which they had been randomised during contact with the researcher. Statistical analysis was undertaken by a member of the research team (FCW) who was blinded to study arm allocation until completion of the initial analyses. The remaining members of the research team were blinded to study arm allocation throughout the duration of the study, with the allocation code not broken until statistical analysis was fully completed.

### Randomisation

The randomisation sequence was generated using a computer, with the system automatically randomising each participant to a study arm and informing the relevant PWP supporting the intervention as well as the service administrator. Minimisation was used to ensure between-group balance by participant age (18–30 years or >31 years), sex (male or female), clinical depression using PHQ-9 score (10–18 or 19–23), current use of antidepressant medication and recruitment site (yes or no) and recruitment site (site 1 or site 2). To maintain concealment, the minimisation algorithm retained a stochastic element.

### Intervention

The intervention adopted in both study arms [17] was based on a written, PWP-supported self-help programme [21]. Following a pragmatic study design [22], delivery of both interventions was aimed at being consistent with the IAPT service delivery protocol for low-intensity cognitive behavioural therapy interventions [21]. Participants received an initial assessment session with the PWP lasting up to 35 minutes, followed by up to 12 support sessions of 25–35 minutes each [21]. The number of subsequent support sessions with the PWP was collaboratively determined between the PWP and the participant. Depending on participant preference, support was provided face to face, over the telephone or through a combination of both methods.

### Behavioural activation

Representing treatment as usual, the BA written self-help intervention was delivered by PWPs in accordance with the BA protocol [21] and implemented as part of the IAPT programme [23, 24]. The PWPs received training to deliver BA during their accredited PWP training programme [21]. The BA protocol [11] was directly transferred into a written self-help format specifically developed and adopted for use within the study [25] (available from the authors upon request). The written self-help booklet was developed alongside separate male and female case study booklets that introduced the participant to a fictional character who was experiencing depression and took the participant through the use of BA with worksheets to be completed.

### Behavioural activation with physical activity promotion

The BAcPAc written self-help intervention booklet was based on the BA protocol [11] implemented in the IAPT programme [23, 24], but it was further informed by findings derived from focus groups with service users and service providers (CP and JRW) and a series of meetings with researchers in the field (PF, AHT, MH, CJG and CP). A full description of the method and design of the intervention can be found elsewhere [17]. The text used within BAcPAc relating to PA promotion was further informed by principles derived from SDT [13, 14]. Self-monitoring is a well-established behaviour change technique [15] and was encouraged by asking participants to measure and record the number of steps walked each day using a pedometer. Intervention-mapping techniques [26] were used to deconstruct the existing BA protocol [21] and inform reconstruction of the BAcPAc written self-help intervention to include PA promotion techniques at various points in the intervention pathway [27]. Consistent with the BA arm, male and female versions of the case studies were developed for each study arm to enhance engagement with the intervention [28].

### Practitioner preparation

All study PWPs had successfully completed an accredited PWP training programme that is based on the Department of Health curriculum [29] developed for the IAPT programme, and they also received additional BA refresher training. PWPs were randomly allocated to the BA or BAcPAc arm by an independent researcher. Six of the original eight PWPs left and were replaced during the course of the study. These individuals included one intervention PWP and one control PWP in site 1 and two intervention PWPs and two control PWPs at site 2. PWPs supporting both interventions received additional training and a training manual [17] specific to the self-help intervention they were supporting and had the opportunity to ask questions. PWPs supporting BAcPAc received additional skill enhancement in use of the BAcPAc materials, PA promotion and motivational interviewing techniques. PWPs in both study arms received routine supervision in accordance with IAPT supervision guidance [29]. Supervisors supporting BAcPAc PWPs were also provided with the BAcPAc intervention self-help booklet and were encouraged to contact a member of the research team to discuss any unresolved questions emerging from supervision or participant sessions.

### Sample size

As this was a pilot study designed to examine methodological and procedural uncertainties, a formal sample size calculation was not performed. A sample size of 80 was derived empirically based on the pilot objectives. Assuming an attrition rate of 20 %, a total sample of 80 participants would give the ability to estimate the attrition that would be seen in a fully powered trial, yielding a 95 % confidence interval (CI) with a width of 18 percentage points. Thirty participants per group (allowing for loss of approximately 20 % of participants over the duration of the pilot) allows estimation of the outcome variance and provides a sufficient pool of participants for qualitative sampling.

### Data collection

Baseline demographic data collected included age, sex, ethnicity, relationship status, smoking status, postcode, number of dependents and age upon leaving education. The following data were gathered at baseline and at the 4-month follow-up time point: 7-day PA recall [30], depression using the PHQ-9 [19] and CIS-R [18], blood pressure (BP) measured using an Omron M7 monitor (Omron Healthcare, Milton Keynes, UK), body mass index (BMI) (weight measured using Omron HN286 digital scale [Omron Healthcare] and height measured using a tape measure), Insomnia Severity Index [31], health-related quality of life (EQ-5D-5L) [32], 36-Item Short Form Health Survey [33], work and social adjustment [34] and a simplified version of the Adult Health and Social Care Service Use [35]. Additionally, to address methodological feasibility questions, IAPT services provided numbers of invitations handed out (site 1) or sent by post (site 2), details of the number of treatment sessions completed between study PWPs and participants and reason for exiting treatment. Accelerometer data were collected at the 4-month follow-up time point only via a waterproof, wrist-worn, triaxial accelerometer (original GENEActiv accelerometer [36]; GENEActiv, Kimbolton, UK), with instructions to wear it continuously for 7 days and nights. To facilitate the assessment of intervention fidelity, study PWPs were asked to digitally record all support sessions with participants (face to face or by telephone). Digital recordings were encrypted and stored securely at the University of Exeter.

### Qualitative data collection

**Fig. 1:**
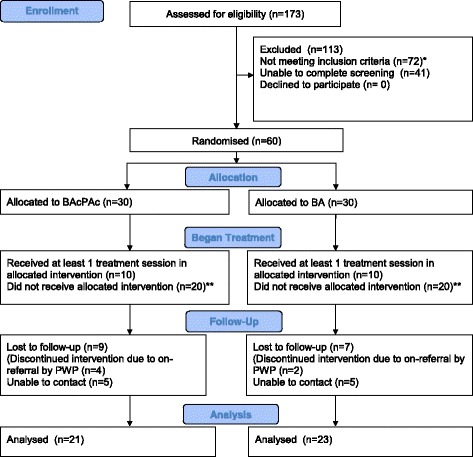
Consolidated Standards of Reporting Trials (CONSORT) flow diagram for combined recruitment at sites 1 and 2 into the behavioural activation with physical activity promotion (BAcPAc) and behavioural activation (BA) study arms. *Exclusions were as follows: 72 in total at the point of screening, 42 (58 %) did not meet depression criteria or primary diagnosis was not depression, 4 (8 %) had changed medication or started new medication in the previous month, 11 (15 %) with confirmed Patient Health Questionnaire indication of risk, 4 (6 %) were unable to walk without aid, 4 (4 %) had difficulties leaving the house or traveling, 3 (4 %) already met activity criteria and 3 (4 %) were <18 years of age. **Forty patients did not receive at least one treatment session (site 1 [*n* = 2], did not attend appointment [*n* = 1]; issues with appointment [*n* = 1]; site 2 [*n* = 38], offered and started different treatment or different psychological wellbeing practitioner [PWP] [*n* = 15]; issues with appointment [*n* = 11], patient dropped out of the study and kept standard treatment appointment [*n* = 5], did not attend [*n* = 6], not suitable or discharged by PWP [*n* = 4]). These data were collected from routine service records

All participants who received at least one therapy session from a study trained PWP, as well as all PWPs trained for the study, were invited to participate in an interview with the researcher. Participants in either the intervention or control arm who did not attend at least one therapy session as randomised were not invited for an interview. Routine service data providing accounts of the patient’s journey for these participants are summarised in Fig. 1. The topic guides for participants (Additional file 3) and PWPs (Additional file 4) were developed with the University of Exeter Lived Experience Group [37]. Notes were taken during the interview, and the main points were summarised and confirmed with the participant following each interview.

### Resource use and costs of intervention delivery

We considered methods of assessing resource use, differences in resource use and costs associated with the intervention delivery. The method used comprised (1) collection of data at the participant level within the pilot trial and (2) combination of resource use data with published unit costs [38] to estimate the mean incremental cost for delivery of BAcPAc versus control (BA alone). Routine service data were requested regarding contact time between PWPs and participants and non–contact time for PWPs (in relation to time required for preparation of sessions). The PWP staff salary grade was assumed to be consistent with that typical for the delivery of BA alone as part of IAPT delivery.

### Accelerometer Data analysis

The raw 100-Hz acceleration data from accelerometers were uploaded to a personal computer using GENEActiv software (version 2.2; ActivInsights, Kimbolton, UK [36]) and converted to a signal vector magnitude using a previously published equation [39]. Averaging values for each 60-second period reduced data further. To be included in the analysis, participants were required to provide data for at least 4 days with ≥10 h of wear (including at least 1 weekend day). Once wear-time compliance had been established, average minutes per day over the valid days in (1) sedentary, (2) light PA, (3) moderate PA, (4) vigorous and (5) moderate and vigorous PA combined intensities were created using published cut-points [39].

### Statistical analysis

In accordance with our pilot study aims, no inferential analyses of the between-group differences in outcomes were conducted. All analyses were performed on an intention-to-treat, complete case basis. Descriptive summaries (means and standard deviations) for outcomes at baseline and 4-month follow-up are reported. The proportions of participants lost to follow-up at 4 months, overall and by treatment arm are also reported, along with 95 % CIs. All analyses were performed using Stata 12 software (StataCorp, College Station, TX, USA).

### Intervention fidelity analysis

The analysis was intended to be performed according to the planned protocol [17]. However, there were not enough data available to allow meaningful intervention fidelity analysis.

### Qualitative data analysis

Interview data from participants receiving at least one treatment session (BAcPAc or BA) and from study PWPs were transcribed verbatim by an independent transcription service. Allocation concealment of participants to study arms was maintained throughout the study and analysis to ensure researchers were not aware which participant had been allocated to which study arm. Thematic analysis [40] was carried out for participant and PWP interviews separately to describe the data and identify relationships and patterns within it. Initial codes were identified during familiarisation with interview data. Transcripts were then coded using the NVivo 10 platform (QSR International, Doncaster, Australia) [41] for each interview in turn, and codes were sorted into overarching themes based on the research questions. The thematic framework was developed in an iterative manner as interviews were coded and then sorted into overarching themes based on the research questions. The thematic framework was refined as coding progressed and checked by revisiting the original data recorded in interview notes. A second researcher (KR) analysed a sample of five participant transcripts (three BAcPAc participant interviews and two BA participant interviews) and four PWP transcripts (two BAcPAc, one site 1 and one site 2; and two BA, both site 2) using the same method. The themes were discussed, and final themes were agreed upon.

### Ethical approval

Ethical approval was granted by the NHS Trust South West Regional Ethics Committee (Research Ethics Committee reference number REC/SW/0291), and research and development approval was received from the Royal Devon and Exeter NHS Foundation Trust. The sponsor was the University of Exeter.

## Results

### Feasibility of recruitment

*Recruitment* is defined as all procedures up to the point of, and including, randomisation of eligible participants. Details of the recruitment procedures at each site are provided in Additional files 1 and 2.

A Consolidated Standards of Reporting Trials (CONSORT) [42] diagram showing recruitment flow into the study, alongside numbers and reasons for exclusions from both sites, is shown in Fig. 1. At site 1, 50 potential participants were invited in person. Of those invited, 15 responded (30 %) and 14 (93 %) were assessed for eligibility. Of those assessed for eligibility, three were not eligible (20 %) and eight (53 %) were eligible and randomised. At site 2, of the 1952 potential participants invited by post, 171 responded (8.8 %) and 159 of these (93 %) were assessed for eligibility. Of those assessed for eligibility, 107 (63 %) were not eligible and 38 were excluded because they had already started treatment within the IAPT service before screening and baseline data collection could be completed. The remaining 52 potential participants were eligible and randomised (2.7 % of those invited and 30.4 % of those who responded). Of the 132 people screened across both sites, 47 were excluded (36 %), because diagnostic criteria for depression were not met.

Follow-up data were collected for a total of 44 (73 %) of 60 randomised participants, with 16 (27 %; 95 % CI 16–40 %) lost to follow-up. In the BA arm, 7 (23 %) of 30 (95 % CI 10–42 %) participants were lost to follow-up. In the BAcPAc arm, 9 (30 %) of 30 participants were lost to follow-up (95 % CI 15 %; 49 %). Of 44 participants, 4 (9 %) disclosed their study arm status during the 4-month follow-up appointment. A service administrator disclosed one participant’s study arm status at the time the participant was receiving treatment.

### Feasibility of data collection

#### Demographic and outcome data

A summary of the assessment data collected during the study is presented in Tables 1, 2, 3 and 4. There were some baseline differences in demographic factors (e.g., sex, marital status), but not for other variables. Only 11 participants (37 %) at baseline and 9 (30 %) at the 4-month follow-up assessment provided data for BMI and BP. BMI and BP data are therefore not presented. Accelerometer data were collected for 42 (70 %) of 60 participants at the 4-month follow-up assessment. Eleven participants (26 %) had missing data (did not return the accelerometer, monitor malfunction and/or corrupt files), and three (7 %) were excluded from analysis owing to failure to meet minimum wear-time criteria. Descriptive data are reported for the 28 (47 %) participants with usable data.

**Table 1:** Baseline demographics

	BA (*n* = 30)	BAcPAc (*n* = 30)
Sex, *n* (%)		
Male	13 (43.3)	18 (60.0)
Female	17 (56.7)	12 (40.0)
Age mean (SD)	44.0 (14.7)	44.8 (13.8)
18–30 yr, *n* (%)	6 (20.0)	6 (20.0)
31+ yr, *n* (%)	24 (80.0)	24 (80.0)
Ethnicity (nationality), *n* (%)		
White (British)	30 (100)	30 (100)
Other	0 (0)	0 (0)
Relationship status, *n* (%)		
Married or cohabiting	21 (70.0)	12 (40.0)
Single, divorced, widowed or separated	9 (30.0)	18 (60.0)
Living with children aged <16 yr, *n* (%)		
No	20 (66.7)	21 (70.0)
Yes	10 (33.3)	9 (30.0)
Current smoker, *n* (%)		
No	20 (66.7)	21 (70.0)
Yes	10 (33.3)	9 (30.0)
Age upon leaving full-time education, *n* (%)		
15–17 yr	21 (70.0)	11 (36.7)
18–20 yr	4 (13.3)	10 (33.3)
21 yr and older	5 (16.7)	9 (30.0)
PHQ-9 score, *n* (%)		
10–18	20 (66.7)	20 (66.7)
19–23	10 (33.3)	10 (33.3)
Centre, *n* (%)		
Site 1	4 (13.3)	4 (13.3)
Site 2	26 (86.7)	26 (86.7)

*BA* behavioural activation, *BAcPAc* behavioural activation with physical activity promotion, *PHQ-9* Patient Health Questionnaire [18]

**Table 2:** Patient questionnaires: psychological status and quality of life

	Baseline		4-mo follow-up	
	BA (*n* = 30)	BAcPAc (*n* = 30)	BA (*n* = 23)	BAcPAc (*n* = 21)
CIS-R, mean (SD)	29.0 (7.3)	27.2 (7.4)	16.7 (9.2) (*n* = 22)	19.3 (10.5) (*n* = 21)
CIS-R primary diagnostic category, *n* (%)				
Mild depression	6 (20.0)	4 (13.3)	2 (9.1)	1 (4.8)
Moderate depression	16 (53.3)	16 (53.3)	2 (9.1)	4 (19.0)
Severe depression	8 (26.7)	10 (33.3)	1 (4.5)	3 (14.3)
No diagnosis	0 (0)	0 (0)	7 (31.8)	5 (23.8)
Other diagnosis	0 (0)	0 (0)	10 (45.5)	8 (38.1)
PHQ-9,^a^ mean (SD)	16.1 (3.8)	16.8 (3.8)	10.1 (5.8)	10.7 (5.7)
WSAS, mean (SD)	23.1 (8.0)	24.6 (7.4)	16.2 (8.3)	16.7 (13.0)
EQ-5D-5L, mean (SD)	0.59 (0.2)	0.61 (0.2)	0.63 (0.2)	0.67 (0.2)
EQ-5D-5L rating (0–100), mean (SD)	54.5 (19.5)	54.6 (17.6)	63.6 (19.2)	57.0 (24.3)
SF-36, mean (SD)				
Physical functioning	59.0 (30.0)	75.8 (24.1)	59.3 (31.2)	76.0 (26.5)
Role-physical	46.0 (27.0)	63.8 (31.4)	48.4 (28.2)	70.8 (30.1)
Bodily pain	49.2 (24.9)	61.7 (25.1)	52.0 (25.0)	65.6 (26.5)
General health	38.9 (22.9)	38.5 (18.7)	41.3 (18.6)	45.3 (23.3)
Vitality	22.5 (14.2)	21.3 (13.5)	32.3 (18.4)	30.7 (18.4)
Social functioning	45.8 (23.3)	41.7 (27.5)	55.4 (21.6)	58.3 (33.2)
Role-emotional	47.5 (21.7)	42.5 (25.5)	56.9 (23.3)	57.5 (36.0)
Mental health	36.3 (14.4)	37.5 (14.0)	51.7 (15.6)	49.0 (24.5)

*BA* behavioural activation, *BAcPAc* behavioural activation with physical activity promotion, *CIS-R* Clinical Interview Schedule–Revised [18], *EQ-5DL* [32], *PHQ-9* Patient Health Questionnaire [19], *SF-36* 36-Item Short Form Health Survey [33], *WSAS* Work and Social Adjustment Scale [34]

^a^All patients were above PHQ-9 threshold for depression at baseline

**Table 3:** Physical activity

	Baseline		4-mo follow-up	
	BA (*n* = 30)	BAcPAc (*n* = 30)	BA (*n* = 23)	BAcPAc (*n* = 21)
Self-reported minutes of moderate physical activity, mean (SD)	19.5 (43.1)	9.7 (13.2)	18.0 (23.7)	7.6 (8.9)
Median (IQR)	1.8 (0.0–15.5)	4.6 (0.0–11.9)	10.7 (1.4–22.1)	6.7 (0.0–10.9)
Self-reported minutes of moderate plus vigorous^a^ physical activity, mean (SD)	19.7 (43.1)	9.8 (13.2)	18.8 (24.0)	9.1 (10.2)
Median (IQR)	1.8 (0.0–16.1)	4.6 (0.0–11.9)	10.7 (1.4–22.1)	8.6 (0.0–13.2)
Accelerometer average minutes per day of sedentary activity, mean (SD)	N/A	N/A	393 (177) (*n* = 13)	373 (165)(*n* = 15)
Accelerometer average minutes per day of light physical activity, mean (SD)	N/A	N/A	339 (170) (*n* = 13)	360 (186) (*n* = 15)
Accelerometer average minutes per day of moderate and vigorous physical activity, mean (SD)	N/A	N/A	130 (89) (*n* = 13)	168 (117) (*n* = 15)

*BA* behavioural activation, *BAcPAc* behavioural activation with physical activity promotion, *IQR* interquartile range, *N/A* not applicable, *SD* standard deviation

^a^At baseline, one participant in the BA arm and one in the BAcPAc arm had >0 minutes of vigorous physical activity. At 4-mo follow-up, three participants in the BA arm and five in the BAcPAc arm had >0 minutes of vigorous physical activity

**Table 4:** Resource use (PWP time) for delivery of interventions and estimated costs for PWP time

Data reported	BA (*n* = 7)	BAcPAc (*n* = 8)	Total (*n* = 15)
Total number of contacts	18	27	45
Number of face to face contacts	7	17	24
Number of telephone contacts	11	10	21
Mean contacts total	2.60	3.38	3.00
Mean contacts face to face	1.00	2.12	1.60
Mean contacts by telephone	1.6	1.25	1.4
Total contact time (min)	685	835	1520
Mean contact time (min/participant)	98	104	101
Mean contact time face to face (min/contact)	42.86	31.18	34.58
Mean contact time by telephone	35	30.5	32.86
Mean estimated cost for delivery of intervention^a^	£156.80	£166.40	£161.60

*BA* behavioural activation, *BAcPAc* behavioural activation with physical activity promotion, *PWP* psychological wellbeing practitioner

^a^PWP unit cost (per hour) is based on behavioural activation delivered by a non-specialist, assumed to be £96/h (excluding qualification cost) of face-to-face contact [36]. The unit cost is assumed to apply to face-to-face and telephone contact time

Owing to the large geographical area covered by the study, telephone baseline data collection appointments, with the possibility of face-to-face appointments if required, were offered to participants. The majority of baseline data were collected over the telephone. Follow-up data at 4 months were collected with a combination of telephone and face-to-face appointments or just face-to-face appointments to allow the researcher to hand an accelerometer to participants.

Follow-up data collection was not possible for 16 (27 %) of 60 of participants. Follow-up rates often reflected the fact that participants had not received any treatment from the service since randomisation. At the time of 4-month follow-up, seven participants refused to wear the accelerometer. Those who gave reasons reported not liking the masculine look of it (participant 109), wanting to avoid explaining it to friends and/or family (participant 227), not wanting to wear it 24 h/day (participant 238) and not wanting to wear it in addition to a watch (participant 246).

#### Qualitative data

We interviewed 9 study PWPs and 15 participants who had received at least 1 treatment session as randomised. The characteristics of those interviewed can be found in Tables 5 and 6. Interviews with participants ranged between 20 and 60 minutes, and those with PWPs ranged between 17 and 40 minutes.

**Table 5:** Characteristics of interviewed psychological wellbeing practitioners

PWP ID	Sex	Allocation	Site	Delivered randomised intervention for a minimum of 1 treatment session
PWP 101	Female	BA	1	Yes
PWP 102	Female	BAcPAc	1	No
PWP 103	Female	BAcPAc	1	No
PWP 104	Female	BAcPAc	1	Yes
PWP 201	Female	BA	2	Yes
PWP 202	Male	BA	2	Yes
PWP 203	Male	BA	2	Yes
PWP 204	Female	BAcPAc	2	Yes
PWP 205	Female	BAcPAc	2	No

*BA* behavioural activation, *BAcPAc* behavioural activation with physical activity promotion, *PWP* psychological wellbeing practitioner

**Table 6:** Characteristics of interviewed participants

Participant ID	Site	Sex	Age (yr)	Allocation
102	1	Male	54	BA
103	1	Female	57	BA
104	1	Male	30	BA
107	1	Female	53	BA
109	1	Female	67	BA
210	2	Male	50	BA
233	2	Male	42	BA
238	2	Female	60	BA
239	2	Male	42	BAcPAc
201	2	Female	60	BAcPAc
211	2	Female	38	BAcPAc
212	2	Female	39	BAcPAc
224	2	Female	61	BAcPAc
227	2	Male	39	BAcPAc
246	2	Male	57	BAcPAc

*BA* behavioural activation, *BAcPAc* behavioural activation with physical activity promotion

#### Intervention fidelity

We collected recorded sessions from two of six BAcPAc PWPs (one at each site), as these were the PWPs who recorded sessions with BAcPAc participants. Only one of these PWPs provided sequential recordings. Meaningful analysis could not be performed, and so was not performed, owing to (1) the small number of participants who received the intervention and (2) only one PWP providing data that could be analysed.

### Estimate of outcome variance to inform sample size of a future phase III randomised controlled trial

The standard deviations derived from these data are similar to published data from larger studies [43]. The minimum clinically important difference for PHQ-9 (the primary outcome for the potential RCT) has been established to be 2.6 with a standard deviation of 5.4 [43]. Using 90 % power and a two-sided significance threshold of 0.05, with attrition of 30 %, the required sample size would be 132 participants per arm, or with 40 % attrition the required sample size would be 154 participants per arm. Using 80 % power and 30 % attrition, 99 participants per arm would be required.

### Estimate of resources and costs needed to deliver the intervention

Data on contact time for PWPs, plus number and type of session, for delivery of BA or BAcPAc were collected from 15 participants in the trial. The mean number of contacts was 2.6 in the BA group (*n* = 7) and 3.3 in the BAcPAc group (*n* = 8), with lower numbers of contacts in both arms than expected for typical BA [38]. Data on resource use indicate that the costs for delivery of the BAcPAc intervention and the comparator are similar, with BAcPAc having a higher mean number of contacts but the overall contact time being similar between arms, as seen in Table 4.

### Qualitative findings

Analysis of interviews provided important information in relation to the specific aims of the study: (1) feasibility of PWPs’ inviting participants, (2) patients’ acceptance and understanding of study procedures and (3) feasibility and acceptability of the intervention. To provide further evidence for qualitative findings, a larger selection of quotes is provided in Additional file 5.

#### Feasibility of psychological wellbeing practitioners’ inviting participants

Although the target of 80 randomised people with depression was not met, we recruited 75 % of this number. Recruitment was much lower at site 1, as substantially fewer than expected invitations were handed out by PWPs. When reasons for the low number of people invited by PWPs at site 1 were explored, important issues included concerns about the impact of inviting participants on PWPs’ working procedures, difficulties with finding time to invite patients within the initial assessment, and PWP preferences for other treatments for depression.

#### Difficulties in psychological wellbeing practitioners’ adapting to recruitment procedures

PWPs at site 1 were reluctant to invite people into the study, as they felt this could cause difficulties in managing their caseload. PWPs were asked to invite people with depression to take part in the study during the assessment appointment and before treatment started. PWPs explained they would normally arrange the first support session with the patient at the end of the initial assessment. However, uncertainty about whether a patient would participate in the study or if an invited participant would start to see a different PWP when randomised resulted in the PWPs’ feeling they had less control than usual over scheduling the first support session, making waiting times less easy to control.

*PWP 104:* ‘I think it is having that person in your caseload without knowing what’s happening to them, and with the PWP caseload people can get lost, so it’s really about making sure you are not losing track of where that person is’.

#### Difficulties with making time to invite potential participants at initial assessment

PWPs spoke of difficulties with completing all the components associated with a low-intensity assessment session [21] with a full discussion of all the treatment options (including BA) within their initial 35-minute appointment.

*PWP 103:* ‘I never had time to discuss that [BA] in the first assessment, getting all of that in within the 35–40 minutes was just not practical. BA would have been discussed in treatment session 1, if they opted for one to one’.

#### Psychological wellbeing practitioners’ preferences for other treatments and their underuse of behavioural activation

PWPs often recommended other treatments for people with depression, based upon their own views of BA and expectations of an individual patient’s ability to engage with BA, and this affected the number of individuals they invited to take part in the study. Analysis of routine service data collected at the end of the study identified that only 2 % of people with depression were receiving BA (59 of 2660) and 98 % (2601 of 2660) were receiving other treatments.

*PWP 102:* ‘Because people [PWPs] have different perspectives, don’t they, and what might help somebody, … some therapists focus more on the cognition, and some therapists focus more on the behavioural part, don’t they?… It’s just selling it to the patient that’s the key aspect; that’s the difficulty with BA’.

Preferences for other treatments affected not only the number of individuals invited but also the number of randomised people who went on to receive at least one BA treatment session, as shown in Fig. 1. This is discussed further in the ‘Feasibility of the intervention’ section below.

#### Participants’ understanding and acceptability of study information and research procedures

Of those participants interviewed, none reported a problem with understanding the study information or its explanation at the screening appointment. One participant thought the written study information material was too long. None of the participants reported an issue with randomisation or the allocated treatment they received. No data were collected from people who did not respond to an invitation or who did not complete recruitment screening.

#### Feasibility of the intervention

Only two of the six trained BAcPAc PWPs (one in site 1 and one at site 2) delivered the BAcPAc intervention (one of whom saw 8 of 10 BAcPAc recipients). As illustrated by the CONSORT [37] diagram in Fig. 1, 10 participants (33 %) in each arm attended the initial treatment session as randomised. It would appear on the basis of the recruitment figures that sending out invitations by post from site 2 yielded higher recruitment, but unfortunately many of those randomised from site 2 who were allocated to either arm did not receive the intervention. This was due primarily to pressures created within the service by sudden, unexpected staff shortages.

#### Staff attrition: randomised participants’ not seeing study psychological wellbeing practitioners

An unprecedented reduction in PWP numbers at site 2 during the study that were due to sickness and staff leaving resulted in service administrators’ having difficulties arranging appointments for randomised patients. As shown in Fig. 1, routine service data collection revealed that 16 of 38 participants had problems keeping their first BAcPAc treatment appointment or kept their routine appointment (given at the start with the invitation letter by the service) rather than attending the new appointment they had been given with a study PWP. Reduced numbers of PWPs across the service put pressure on administrators to prioritise rearranging appointments for waiting patients quickly rather than ensuring that participants were seen by the correct study PWPs. This adversely affected uptake of the randomised allocation at Site 2:

*PWP 203:* ‘It was really unfortunate timing in a way that when the study was going on we had some real staffing issues.… We were ridiculously overstretched, and our waiting lists were growing and growing, but when we put something else on top, like an involvement with a study, sometimes it is hard to pull it off with the day-to-day stuff. Like, we can’t have things like trying to keep slots open, reserved for BAcPAc, when [owing to staff shortages] some of our PWPs are booking 40 appointments a week’.

#### Low uptake of behavioural activation or BAcPAc as a treatment following randomisation

It was expected that, once they agreed to take part, participants would start the intervention consistent with their allocated randomisation. However, 15 of 38 randomised participants at site 2 started a different treatment instead, as shown in Fig. 1. The PWPs’ explanation of this was that, even though participants had depression confirmed as the primary diagnosis based on the CIS-R at the time of screening, some wanted to be treated for anxiety rather than depression. Other participants were reluctant to try BA.

*PWP 204:* ‘Obviously, they don’t always know what’s the best for them, and a lot of people will then say what problem is having the most impact at that moment’.

#### Acceptability of the intervention

*Acceptability* refers to the appropriateness and relevance of the intervention for recipients and PWPs [44]. Specific focus was placed upon the acceptability of PA promotion as part of the rationale for recovery and the acceptability of the BAcPAc booklets.

#### Promoting physical activity was acceptable to patients

On the basis of past experience with treating patients interested in increasing their activity levels, PWPs in both study arms indicated their familiarity with promoting PA as appropriate to patients using BA and believed PA to be helpful for recovery. BAcPAc was therefore not considered to be a radical change to their existing practice.

*PWP 204:* ‘Generally they, [the patients] get it fairly easily. I mean, actually something I find with general BA is that a lot of people, when you talk about activity, think that you just mean physical activity anyway’.

BAcPAc participants were also open to increasing PA to return to their more active selves.

*Participant 211:* ‘So, it was actually pushing myself to go out and do more active things. It was something I did before, but actually being able to do it again…, I come back feeling a lot better in myself’.

Of the seven participants interviewed in the BAcPAc arm, five mentioned increasing walking, and of these, three joined a weekly walking group. Other specific activities that were restarted included dancing, gardening and swimming. Getting out more was associated with more PA for many, and often this incorporated walking. However, three of the eight interviewed in the BA arm also spoke of an increase in PA through walking. Other activities included joining a gym and gardening.

Not all engaged with the idea of increasing PA during the trial.

*Participant 212:* ‘Even getting out of the house was a big big problem for me, so I may not have been as receptive to it as perhaps I could’.

#### Benefit of self-monitoring to increase physical activity

Both PWPs delivering the intervention in the BAcPAc arm talked about liking the pedometers as a self-monitoring tool and promoted pedometers to participants. Of the seven participants interviewed in the BAcPAc arm, three specifically mentioned enjoying using a pedometer to help increase and monitor their PA.

*PWP 204:* ‘Yeah, people were really keen on that [pedometers]. I think everyone I suggested it to wanted one, so that would be three or four people’.

*Participant 104:* ‘I was given a pedometer. That kind of helped me to focus more on walking every day. So that was quite helpful, and it makes you kind of aware of it, which kind of helps’.

The diaries seemed to be one of the most useful tools in the booklets.

*Participant 102:* ‘Writing it down in the diary and actually categorising what’s going on at each point I would probably say [was] the one real thing that was useful out of the therapy’.

#### Acceptability of the booklets

PWPs generally approved the booklets and liked the layout, use of illustrations and clarity of the text and the written tasks.

*Researcher:* ‘Can you think of anything that would improve the booklet, like size, length, layout or the pace of what you have been explained’?

*Participant 211:* ‘No. It was all really clear, and there was enough space to write things in’.

Several participants commented on being motivated enough to continue to engage with the booklets on their own outside the support sessions and completing the tasks within the booklet.

*Participant 103:* ‘I liked the workbooks, that you could go away and it was self-led. I think I could have done them on my own, but I do think the very fact I had an appointment with somebody did focus my mind’.

One participant (BAcPAc arm) spoke of stopping treatment after two sessions but continued to use the self-help intervention to plan activities and monitor PA using the diary.

*Participant 103:* ‘I used walking as my activity. I bought a pedometer and just kept upping my steps all the time. And just being out walking’.

*Researcher:* ‘And did you feel that was a result of these workbooks prompting you to do that’?

*Participant 103:* ‘Yeah, definitely’.

Unfortunately, BAcPAc PWPs’ views of the length of the booklets, plus the burden of carrying additional paperwork, impacted whether booklets were handed to all participants.

*PWP 102:* ‘I think they were really well produced, but I think maybe the amount of information, it was quite a lot [for patients].… I must admit, because I have so much to carry as a PWP, it was a bit too much’.

*PWP 204:* ‘I didn’t use the case studies that much, and I didn’t actually give them to some of the later people I’ve worked with, because the people I gave them to early on didn’t read them. I think it was too much’.

#### Remedial actions to boost recruitment during the study

Remedial actions were taken where possible as recruitment issues became apparent. The number of PWPs who were trained to invite participants at site 1 was increased from 4 to 19. This change resulted in an initial small increase in the number invited. Several GPs referring into the services were adjusting medication doses upon referral into the IAPT service. Given that this was a pilot RCT and that calculation of effect size was not a study aim, it was decided to remove the exclusion criteria related to change in antidepressant medication 1 month before screening, but to continue to record all changes in medication. This change also resulted in a small increase in recruitment.

## Discussion

A pilot trial was undertaken to identify the feasibility and acceptability of BAcPAc delivered within routine IAPT services to people with depression, to test trial methodology and procedures, to estimate outcome variance and to estimate resource use and costs to inform progression to a fully powered RCT. We achieved recruitment of 60 (75 %) of the target of 80 participants and collected follow-up data from 44 (73 %) of 60 participants and accelerometer data from 42 (70 %) of 60 participants. Although 30 % of participants did not participate in a follow-up appointment or accept wearing an accelerometer, adherence to the predetermined minimum accelerometer wear time for inclusion in this study was 93 % (39 of 42) for those who provided data. This is in accord with other data showing high adherence to use of waterproof wrist-worn accelerometers [45, 46]. The attrition rate of 27 % was comparable with attrition in other trials of depression and exercise, which range from 0 to 44 % with a mean of 19.9 % [6].

The challenge of recruiting to target in existing front-line services when clinical priorities and contractual issues come first has also been observed in other studies [47, 48]. The main challenges in recruitment and participants’ completing the study were staff absence and attrition, which have been recognised as areas of concern in the PWP workforce [49]. Furthermore, staff attrition at site 1 was due to service reorganisation, resulting in both the service manager and other key staff at site 1 leaving just before the study started and causing delays in gaining approvals, in recruitment and in retraining the study PWPs. The staff shortages placed great demands on existing clinicians. The staff shortages in the services increased patient waiting times and resulted in some participants’ being seen by a non-study PWP as attempts to manage patient waiting times became the service’s main concern.

Recruitment and data collection also proved challenging owing to the constraints of the IAPT performance targets set for services. Both services operated within a maximum waiting time of 28 days from receipt of referral to initial assessment, which had been set as a key performance indicator for the IAPT programme [50]. Consequently, if recruitment procedures could not be completed before the initial assessment (often provided within much less than 28 days), patients kept their appointments for normal care and were unable to participate in the research.

Finally, recruitment was further stretched as a result of two additional, but unexpected, events that strained our resources available for delivering the trial per protocol. First, a single recruitment site near the research centre was no longer available, and collaborating recruitment services up to 45 miles away was required. Second, a senior member of the research team with knowledge of the BA intervention had a serious 6-month illness during the recruitment period. This presented a challenge to supervision provided to the study PWPs to support intervention delivery, alongside planned liaison with the service management.

### Strengths and limitations

This was the first study we are aware of to develop and investigate the feasibility of a supported self-help intervention combining BA and PA promotion for people with depression. The study aims of seeking to address uncertainties about the feasibility of recruitment from IAPT services, feasibility of study and data collection procedures were achieved. Overall, participants were positive about the self-help booklets and found self-monitoring with the assistance of diaries and pedometers useful, and they thought group-based physical activities such as walking to be useful. Furthermore, this is one of the few studies to explicitly address feasibility issues associated with running an RCT within services set up within the IAPT programme, which is currently being implemented across England. We collected qualitative and quantitative data that identified barriers to recruitment, to study procedures and the feasibility and acceptability of the intervention and suggested possible solutions to the problems discovered. It would also have been helpful to interview randomised participants who did not receive treatment as allocated.

Low numbers of participants, particularly those receiving the intended interventions, resulted in several uncertainties relating to the intervention. Such low numbers make it difficult to derive an accurate estimate of resource and intervention costs or outcome variance, which resulted in a wide CI for the total proportion lost to follow-up. However, the estimate of the proportion of people who are likely to meet eligibility criteria for the main trial is probably an underestimation due to service pressures and lack of PWP adherence to guidelines regarding therapy selection. Although useful data have been collected and reported, these limitations currently preclude progression to a full-scale RCT using the same methods employed here.

### Implications for planning a future trial

The conduct of this study demonstrates the importance of the pilot phase in assessing the feasibility of an intervention and methodological issues to address before conducting a fully powered RCT [44, 51]. Owing to the current pressures within the IAPT services, finding ways of enabling PWPs to engage with study procedures is recommended. In the present study, inviting potential patients was too much of a burden for PWPs. Having administrators send out invitation letters was more feasible.

Uncertainty remains about several important issues, and further research is required before we progress to a definitive trial:

1. A setting needs to be identified where we can deliver a standardised version of the BA intervention with good quality supervision to prevent treatment protocol deviation. This might incorporate protected research time for clinicians [52].
2. Intervention fidelity for both standard BA and BAcPAc needs to be examined. This might include recording and coding sessions to establish differentiation of content and/or techniques used to promote PA and, within the intervention group, to show how the promotion of PA differed pre- and posttraining. It might also include interviewing practitioners and patients about their experiences during the training and delivery of BAcPAc (in comparison with BA as usual).
3. Trial procedures need to be established that produce increased recruitment and retention rates.
4. We need to explore the variance in outcomes and scale of changes in outcomes for both BA as usual and BAcPAc to enable conduct of a sample size calculation. These issues could be addressed by further feasibility or pilot work, such as an in-service evaluation, to address intervention fidelity issues or an internal pilot study to address the remaining trial feasibility questions.

## Conclusions

This study demonstrates the difficulties of embedding a pilot trial into existing current clinical practice, in this case IAPT services. Staffing issues and service pressures impacted recruitment and made delivery of the procedures and the intervention challenging for the PWPs. Low numbers of people receiving treatment as randomised affected retention at follow-up. However, qualitative data indicated that the intervention and intervention booklets were acceptable to both patients and PWPs, and people with depression accepted the importance of increasing PA as a method to aid their recovery. Furthermore, it was acceptable to use diagnostic tools and to collect data from participants with depression over the telephone. We identified a number of barriers to the feasibility and acceptability of the research procedures and intervention, and specific suggestions are made for further research needed before progression to a definitive RCT.

## Additional files

Additional file 1:
**Recruitment pathway: site 1.** (PDF 106 kb) Additional file 2:
**Recruitment pathway: site 2.** (PDF 101 kb) Additional file 3:
**Interview topic guide for participants.** (PDF 218 kb) Additional file 4:
**Interview topic guide for PWPs.** (PDF 312 kb) Additional file 5:
**Additional quotes derived from interview data.** (PDF 106 kb)

## Abbreviations

BA: behavioural activation
BAcPAc: behavioural activation with physical activity promotion
BMI: body mass index
BP: blood pressure
CI: Confidence interval
CIS-R: Clinical Interview Schedule–Revised
CONSORT: Consolidated Standards of Reporting Trials
GP: general practitioner
IAPT: Improving Access to Psychological Therapies
IQR: interquartile range
PA: physical activity
PHQ-9: Patient Health Questionnaire
PWP: psychological wellbeing practitioner
REC: Research Ethics Committee
RCT: randomised controlled trial
NHS: National Health Service
NIHR: National Institute of Health Research
SD: standard deviation
SDT: self-determination theory
SF-36: 36-Item Short Form Health Survey
WSAS: Work and Social Adjustment Scale
