# Supplementary figures and images for: Combining behavioural activation with physical activity promotion for adults with depression: findings of a parallel-group pilot randomised controlled trial (BAcPAc)
Source: Trials. 2015 Aug 20;16:367. doi: 10.1186/s13063-015-0881-0 (PMC4545876; doi:10.1186/s13063-015-0881-0)

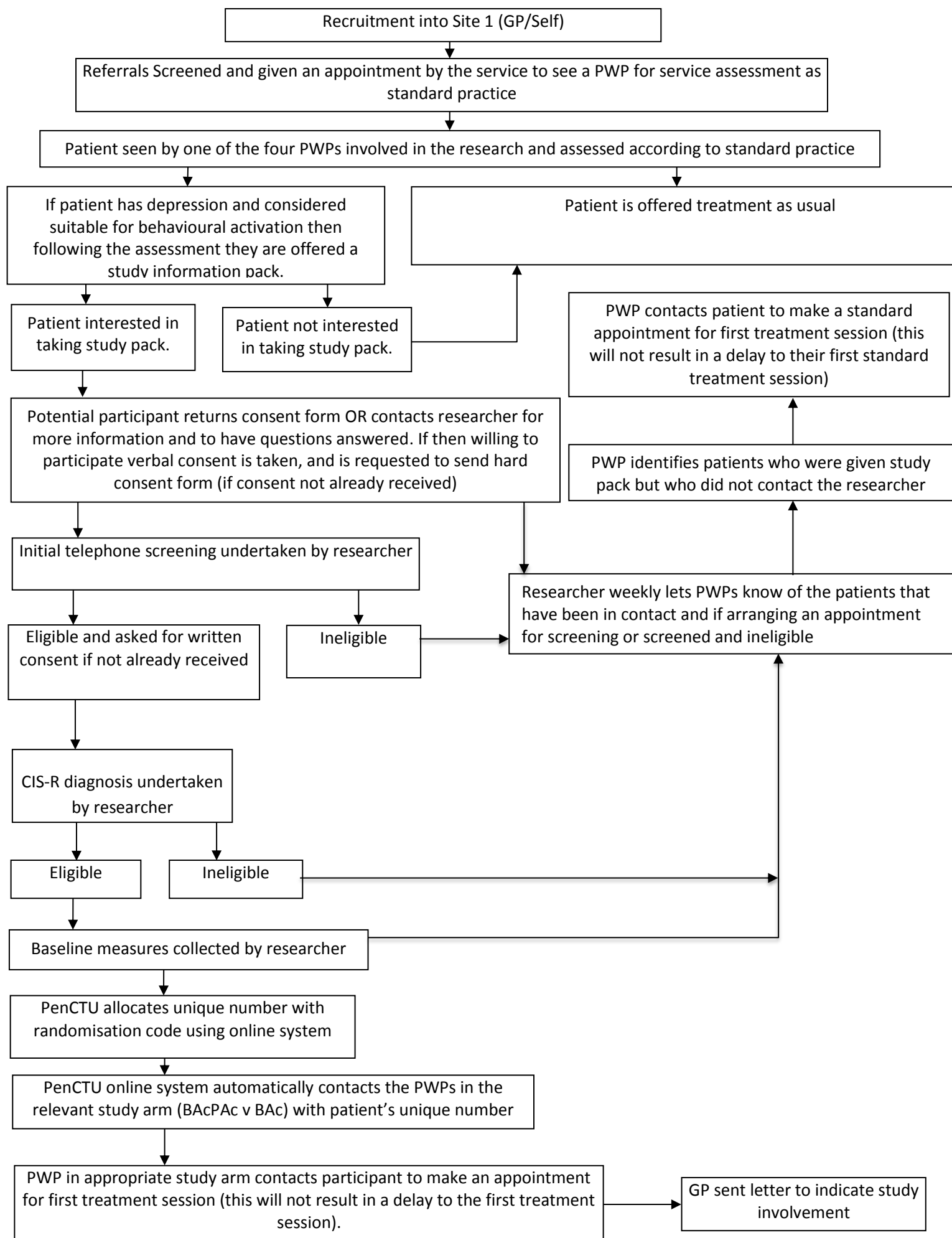

Supplement: Additional file 1: — Recruitment pathway: site 1. (PDF 106 kb) [file 13063_2015_881_MOESM1_ESM.pdf]

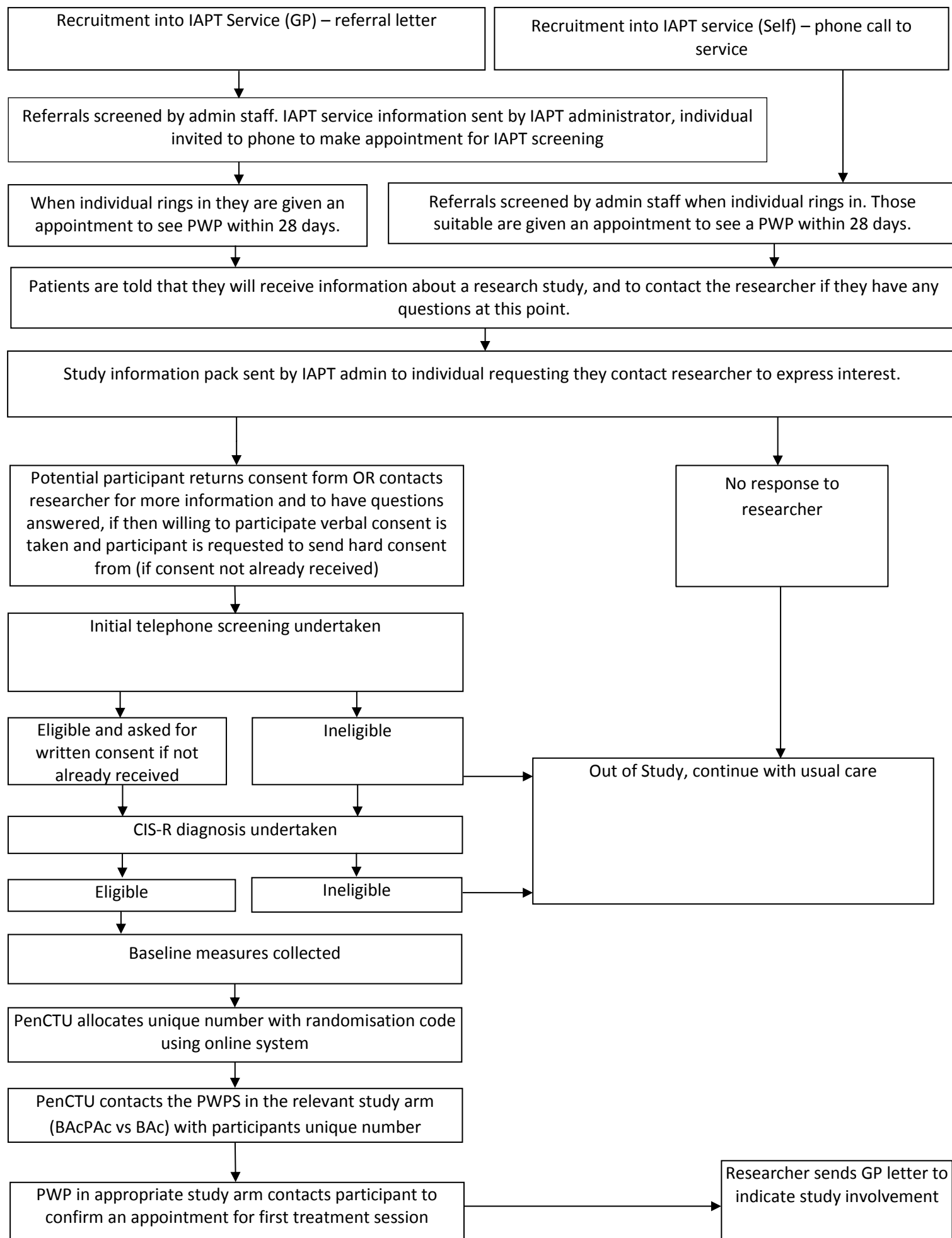

Supplement: Additional file 2: — Recruitment pathway: site 2. (PDF 101 kb) [file 13063_2015_881_MOESM2_ESM.pdf]
