# Supplementary material for: Combining behavioural activation with physical activity promotion for adults with depression: findings of a parallel-group pilot randomised controlled trial (BAcPAc)
Source: Trials. 2015 Aug 20;16:367. doi: 10.1186/s13063-015-0881-0 (PMC4545876; doi:10.1186/s13063-015-0881-0)
Supplement: Additional file 4: — Interview topic guide for PWPs. (PDF 312 kb) [file 13063_2015_881_MOESM4_ESM.pdf]

Topic	Questions	Possible prompts
Opening questions	Tell me a little bit about how you got involved in the study?	How did you hear about the study?
	What were your views of the research?	Prompt: Training opportunity, interest in Physical Activity, interest in research, previous experience, something else?
BAcPAc Delivery	How did working with BAcPAc compare to working with usual BA for depressed patients?	Examples?<br>Did you do anything differently?
	We are interested in why we had difficulties in recruiting many patients who were suitable to receive BA or BAcPAc. Do you have any thoughts on this? How did you decide if someone was suitable for BA/BAcPAc	Suitability for BA/Criteria<br>Protocol/procedures?<br>Difficulties seeing recruited patients?<br>Anything else?
	How did you find talking to the participants about adding physical activity?	Usefulness of the materials (explanations, diaries, self monitoring)<br>Using existing BA skills to negotiate change<br>Overcoming resistance.<br>When in the course of treatment did you have the chance to focus on helping patients to increase their physical activity and why then?
	How did you get on with the regular sessions/phone calls with patients?	Any positive/negative experiences<br>Timing, duration, content, usefulness
	What did you like/not like about the follow up support sessions/phone calls with patients What worked well overall?	Timing, frequency, planned content.<br>Anything else?
	Did not work so well overall?	
Views and experiences of the materials	What were your views of the *'Let's Get Active'* written self-help intervention and case studies?	What did you like/not like?<br>What did you find useful?<br>What could be improved?<br><br>length, style, format, diagrams, diaries.<br>Anything else?
Views and experience of the training	What were your views of the training? (both formal and informal)	What did you like/not like?<br>What did you find useful?<br>What could be improved?<br><br>Location, duration, content.
Experience of research	Did involvement in the study	Handing out invitation packs (SITE 1

procedures including supervision	present any difficulties?<br><br>Did any of the procedures affect the way you dealt with participants?<br><br>Did involvement in the study interfere with care delivery?<br><br>Were there any potentially eligible participants for BAcPAc that you did not introduce to the research study? If so how many, and what were your reasons behind this.	ONLY)<br><br>On-going training<br>Communication with the researcher<br>Ease of: recording the interviews, keeping records, the timing/content/duration of group supervision sessions.<br>Anything else?
Summary	SUMMARISE: Is that about right? Do you have anything that you would like to add?	
