# Supplementary material for: Combining behavioural activation with physical activity promotion for adults with depression: findings of a parallel-group pilot randomised controlled trial (BAcPAc)
Source: Trials. 2015 Aug 20;16:367. doi: 10.1186/s13063-015-0881-0 (PMC4545876; doi:10.1186/s13063-015-0881-0)
Supplement: Additional file 5: — Additional quotes derived from interview data. (PDF 106 kb) [file 13063_2015_881_MOESM5_ESM.pdf]

Feasibility of recruitment

	Low numbers invited at Site 1
Difficulties for PWPs adapting to recruitment procedures	Researcher: “Were there any people who you thought might be eligible but you did not give a pack to?<br>PWP 101: I think there may have been a couple, yeah.<br>“Any reasons that might have been?”<br>“Probably forgetting – although thinking afterwards, Oh that would have been suitable for that study.”<br><br>PWP 104: “I think it is having that person in your caseload without knowing what’s happening to them, and with the PWP caseload people can get lost so it’s really about making sure you are not losing track of where that person is.”<br>… “When I assess somebody, I like to sort of see them [for treatment], it is difficult picking up a person that’s been assessed by someone else. Maybe that’s got something to do with the continuity and everything.”<br><br>PWP 103: “[PWPs] were a little bit uncertain about the time lapse between inviting somebody and then them [potential participants] going onto the research programme, whether they [PWPs] should make another appointment to see them [potential participants] again or, or not, so you will be wondering, are they lost within that process…PWPs might be a bit reluctant to break what they’ve seen as almost they’ve started that therapeutic relationship within the assessment session and said they are suitable for treatment and now we are going, you’re interested in the study so it might be that you are attached to someone else. Some people are reluctant to give over that patient to someone else.”
PWP preferences for other treatments, and underutilisation of BA	PWP 102: “Because [PWPs] have different perspectives don’t they and what might help somebody. The thing is, [with] CBT, as you know, some therapists focus more on the cognition, and some therapists focus more on the behavioural part, don’t they? BA itself can be quite challenging for people, can’t it? So that’s a difficult one sometimes for people to engage with… It’s just selling it to the patient, that’s the key aspect, that's the difficulty with BA.”<br><br>PWP 104: “The clients that presented with depression tended to go into the groups, tended to be happy to go onto the groups. As a service as well and the pressures we were under

as PWPs it was to get as many sort of as we could to get people onto groups…takes off the workload, and in terms of your kind of caseload and your one to ones, you don't have as much of a waiting list because you are putting people onto groups."

PWP 104: "At the end of the assessment you might show them the CBT diagram and discuss with them how it works and I will say we could focus around the thoughts or the behaviour, I don't give any more information than that. And they tend to say, yep I would probably like to focus on the thoughts."

PWP 101: "I think for many people the BA approach does not really work for them."

Feasibility of recruitment

Participants understanding and acceptability of study information and research procedures

Understanding study information and randomisation

Researcher: "So when you got in contact with me we discussed a little bit about what was involved. Was the information about the study at that point clear?"
Pt 102: "Yes"
Did you understand you were going to be randomly put into one of two groups?
"Absolutely, yes."

Researcher: "Were you informed that you would potentially be put into one of two groups for the study?"
Pt 103 "Yes Yes I was."
"How did you feel about that idea?"
"Yeah, I was ambivilent, to be honest, because I just, it did not matter to me which group it was."

Researcher: "You were told about being put into one of two groups, was that information quite clear to you?"
Pt 104 "Yes. I'm kind of aware of how studies are done, so that was expected."

Researcher: "Do you remember we told you you would be put into one of two groups?"
"That's right, yes."
"And did you have any problems with that at all?"
Pt 107: "No, I had no problem at all."

Researcher: "Was it explained well enough?"

Pt 109: “Yes.”
“And what did you think about being put into one of two groups?”
“Yeah yeah, that was fine, yeah.”

Researcher: “And so thinking about when we first chatted, did you understand what was expected of you and did you have all the information you needed?”
Pt 210: “Yes I think so.”
“And in regards to being put into one of two groups, do you remember that was the case.”
“Yes I remember that, that was clear, yeah.”

Feasibility of research procedures

Researcher: “And later we had the screening and data collection. Any issues or problems?”
Pt 102: “No, no, it was fine.”

Researcher: “How was the screening and data collection appointment then?”
Pt 107: “Okay, no problem. I realised that it was something that had to be done, because it was a study.”

Researcher: “And we had to go through some questions over the phone. How was that?”
Pt 109: “No no, that was fine, all straight forward.”
“Anything about those appointments you would have liked to have changed?”
“No, no that was all fine, thank-you.”

Researcher: “How did you find those screening and data collection appointments?”
Pt 210 “Well they were relevent to my needs, I think. And the questions were to the point, mainly yes or no. So yeah, that was fine.”
“Is there anything that you would have liked to have changed about those appointments?”
“Err, face to face is good, I’m not very good over the phone. If I did it again I think I’d rather, I know you came here once, but I think over the phone is not so personable.”

Pt 211: “It was fine. It does take a while to think it through and make sure you have given the right answers, but yeah, it does not take too long so it was fine.”

Pt 212: “Speaking to you felt like it was one of the few occassions where I felt I had been heard.”

Researcher: “Was there anything that you would liked to have changed about those appointments?
Pt 233: No, not really, it was fine over the phone and the appointment in [town name] was fine.”

Feasibility of the intervention

Randomised participants not able to see a study PWP due to staffing issues	PWP 203: “It was really unfortunate timing in a way that when the study was going on we had some real staffing issues. Like the PWPs basically do all the assessments for the team, and we get between 300-400 referrals a month just in our little team. And so all those 300 or 400 people have to be assessed by the PWPs each month. And when we were doing the BAcPAc study we had, what 3 people leave and 2 or 3 go off sick for more than a couple of months, so the PWP quota was pretty much halved, so we were trying to cover that many assessments with half the PWPs, we were ridiculously over-stretched and our waiting lists were growing and growing.”<br><br>PWP 203: “Our little team works from [town W] to [town X], which is 65 miles across, we were spread thinly. We had a couple of PWPs in [town Y] and a couple in [town Z] …so if a patient expresses an interest in [town X], there might not be a PWP that is part of the BAcPAc study.”<br><br>PWP 203: “It is like we were treading water most of the time…we were constantly trying to move things around, juggling staff, changing clinicians, trying to recruit [PWPs], trying to stop people from going off sick, just to keep it functioning as a service, but when we put something else on top, like an involvement with another study, sometimes it is hard to pull it off with the day to day stuff. Like we can’t have things like trying to keep slots open, reserved for BAcPAc, when [due to staff shortages] some of our PWPs are booking 40 appointments a week.”

Acceptability of the intervention

PA was acceptable to patients	PWP 204: “Generally [the patients] get it fairly easily. I mean, actually something I find with general BA is that a lot of people when you talk about activity think that you just mean physical activity anyway.”<br><br>Pt. 211: “So it was actually pushing myself to go out an’ do more active things. It was something I did before, but actually being able to do it again…I come back feeling a lot better in myself.”<br><br>Pt. 201: “It did actually get me to seek a walking group to do walking on a Tuesday. I’ve also got crab pots that I check every day now, so that is another load of walking. If I hadn’t seen the PWP in the first place I wouldn’t even have looked into the walking group, I would have just stayed in my room.”<br><br>Pt. 227: “I’ve done some stuff like gardening, a bit of decorating, its given me the motivation to get up in a morning, get out of the house, not sort of lounge about and feel sorry for myself, it has helped me 300%.”
Benefit of self-monitoring to increase physical activity	PWP 204: “Yeah, people were really keen on [pedometers]. I think everyone I suggested it to wanted one, so that would be three or four people.”<br><br>Pt. 104: “I was given a pedometer. That helped me kind of focus more on going on walking every day. So that was quite a helpful aid because it makes you kind of aware of it, which kind of helps.”<br><br>Pt102: “Writing it down in the diary and actually categorising what’s going on at each point. I would probably say the one real thing that was useful out of the therapy.”<br><br>Pt 107: “The diary, I found was very good come the end, because you can reflect, you can go back and see the bits that made you feel good, and you think, oh I am going to do that again.”<br><br>Pt 211: “They [the books] were really helpful and really practical, and actually working through it step by step and keeping a record of it, that was really useful.”

Acceptability of the booklets - patients	Researcher: "What were your general thoughts about the booklets?<br>Pt 104:"Clear, concise, useful, they generally helped break things down."<br><br>Pt. 224: "I particularly liked the diary thing, especially the double page, it was nice to open it up and see the whole week."<br><br>Researcher: "Can you think of anything that would improve the booklet like size, length, layout or the pace of what you have been explained?"<br>Pt 211: "No. It was all really clear and there was enough space to write things in."<br><br>Pt 103: "I liked the workbooks, that you could go away and it was self-led. I think I could have done them on my own, but I do think the very fact I had an appointment with somebody did focus my mind."<br><br>Pt 109: "When I feel a bit down I can just go back and look at that [book] and take it on board again…I did not get to see a lot of [PWP], because of circumstances on [PWP] part, so I went weeks without seeing anybody, so I did keep looking at the books and think, yeah, you should be getting on with that now."<br><br>Pt 103: "I used walking as my activity, I bought a pedometer and just kept upping my steps all the time. And just being out walking."<br>Researcher: "And did you feel that was a result of these workbooks prompting you to do that?"<br>Pt. 103: "Yeah, definitely."<br><br>Pt 104: "It actually worked really well for me. The book helped me, basically breaking down my day into those categories was very, very helpful. And I keep going back to that now too, and I try to make some time for anything that will give me a bit of pleasure."
PWPs' views of the materials	PWP 204: "The materials are useful because certainly there is more information in the materials than I went through with people, so it is obviously always handy to just direct people so they can read a bit more about it…I didn't use the case studies much and I did not give them to some of the later people I've worked with, because the people I gave them to early on didn't really read them."

PWP 102: “I thought they were nice, they were sort of set out well, good explanations throughout, I did not have any problems with the material itself, I thought that was really good...I think the case studies were quite good, the examples of the case studies were good…But a bit too much material really, and sometimes people might find it a little bit overwhelming…The amount of information it was quite a lot. Sometimes people don't want to engage and do homework do they? If they find it difficult to fill in all the sheets and everything sometimes… Because I must admit, I did find it a little bit, because I have so much to carry myself as a PWP, I felt it was a bit too much.”
